# Genome-wide identification and analysis of the growth-regulating factor family in Chinese cabbage (*Brassica rapa* L. ssp. *pekinensis*)

**DOI:** 10.1186/1471-2164-15-807

**Published:** 2014-09-22

**Authors:** Fengde Wang, Nianwei Qiu, Qian Ding, Jingjuan Li, Yihui Zhang, Huayin Li, Jianwei Gao

**Affiliations:** Institute of Vegetables and Flowers, Shandong Academy of Agricultural Sciences/Shandong Key Laboratory of Greenhouse Vegetable Biology/Shandong Branch of National Vegetable Improvement Center, Jinan, 250100 China; College of Life Science, Qufu Normal University, Qufu, 273165 China

**Keywords:** Chinese cabbage, Expression, Gene family, GRF, Transgenic lines

## Abstract

**Background:**

Growth regulating factors (GRFs) have been shown to play important roles in plant growth and development. GRF genes represent a large multigene family in plants. Recently, genome-wide structural and evolutionary analyses of the GRF gene families in Arabidopsis, rice, and maize have been reported. Chinese cabbage (*Brassica rapa* L. ssp. *pekinensis*) is one of the most important vegetables for agricultural production, and a full genome assembly for this plant has recently been released. However, to our knowledge, the GRF gene family from Chinese cabbage has not been characterized in detail.

**Results:**

In this study, genome-wide analysis was carried out to identify all the GRF genes in Chinese cabbage. Based on the complete Chinese cabbage genome sequence, 17 nonredundant GRF genes, named *BrGRF*s, were identified and classified into six groups. Phylogenetic analysis of the translated GRF protein sequences from Chinese cabbage, Arabidopsis, and rice indicated that the Chinese cabbage GRF proteins were more closely related to the GRF proteins of Arabidopsis than to those of rice. Expression profile analysis showed that the *BrGRF* genes had uneven transcript levels in different organs or tissues, and the transcription of most *BrGRF* genes was induced by gibberellic acid (GA3) treatment. Additionally, over-expression of *BrGRF8* in transgenic Arabidopsis plants increased the sizes of the leaves and other organs by regulation of cell proliferation.

**Conclusions:**

The data obtained from this investigation will contribute to a better understanding of the characteristics of the GRF gene family in Chinese cabbage, and provide a basis for further studies to investigate GRF protein function during development as well as for Chinese cabbage-breeding programs to improve yield and/or head size.

**Electronic supplementary material:**

The online version of this article (doi:10.1186/1471-2164-15-807) contains supplementary material, which is available to authorized users.

## Background

Growth regulating factors (GRFs) are plant-specific proteins that play important roles in regulating plant growth and development. A GRF gene was first identified in rice where it encodes a protein that functions in regulating gibberellic acid (GA)-induced stem elongation [1].

The deduced protein products of GRF genes contain two conserved domains in the N-terminal regions, the QLQ and WRC domains. The QLQ domain interacts with GRF interacting factors (GIF) and the resulting complex acts as a transcriptional co-activator [2], while the WRC domain comprises a functional nuclear localization signal (NLS) and a zinc-finger motif that functions in DNA binding [3].

Currently, the GRF gene family consists of nine *Arabidopsis thaliana* genes [3], 12 *Oryza sativa* genes [4], 14 *Zea mays* genes [5], and 10 *Brachypodium distachyon* genes [6]. In these plants, the GRF genes are strongly expressed in actively growing and developing tissues, such as shoot tips, flower buds, and immature leaves, but weakly expressed in mature tissues or organs. GRF genes have been reported to act as positive regulators of leaf size through the promotion and/or maintenance of cell proliferation activity in leaf primordia [2, 3, 7, 8]. In *Brassica napus*, *GRF2* was found to enhance seed oil production by regulating cell number and plant photosynthesis [9]. GRF genes may act by regulating cell proliferation through the suppression of *KNOX* gene expression [10], which inhibits GA biosynthesis in the S-adenosyl methionine (SAM) cycle by down-regulating the key biosynthetic gene GA20 oxidase [11–15], or by controlling the level of GA2 oxidase 1, which degrades GA [16]. Recently, many studies have reported the involvement of GRF genes in the regulation of flower development [17–19].

Chinese cabbage *(Brassica rapa* L. ssp. *pekinensis)* is one of the most important vegetables for agricultural production in Asia and, although it originated in China, it is increasingly popular in other countries around the world. Yield enhancement is now one of the critical targets of plant breeding programs focused on genetic improvement. Understanding the functions of GRF genes in regulating organ size in Chinese cabbage will achieve this objective. However, knowledge about the molecular features of GRF genes in Chinese cabbage is limited. Therefore, identifying and characterizing GRF genes in Chinese cabbage is of great interest.

In this study, 17 putative GRF genes of Chinese cabbage were identified from the Brassica database (http://brassicadb.org/brad/) [20]. The expression patterns of these *BrGRF*s and the molecular features of the translated BrGRF proteins were analyzed, and the function of BrGRF8 was studied further. The results indicated that GRF genes had higher expression levels in immature organs or tissues than in mature ones, and their transcription was induced by gibberellic acid (GA3). Further analysis showed that cell proliferation was enhanced in transgenic plants.

## Results

### Identification of the *BrGRFs*

**Figure 1:**
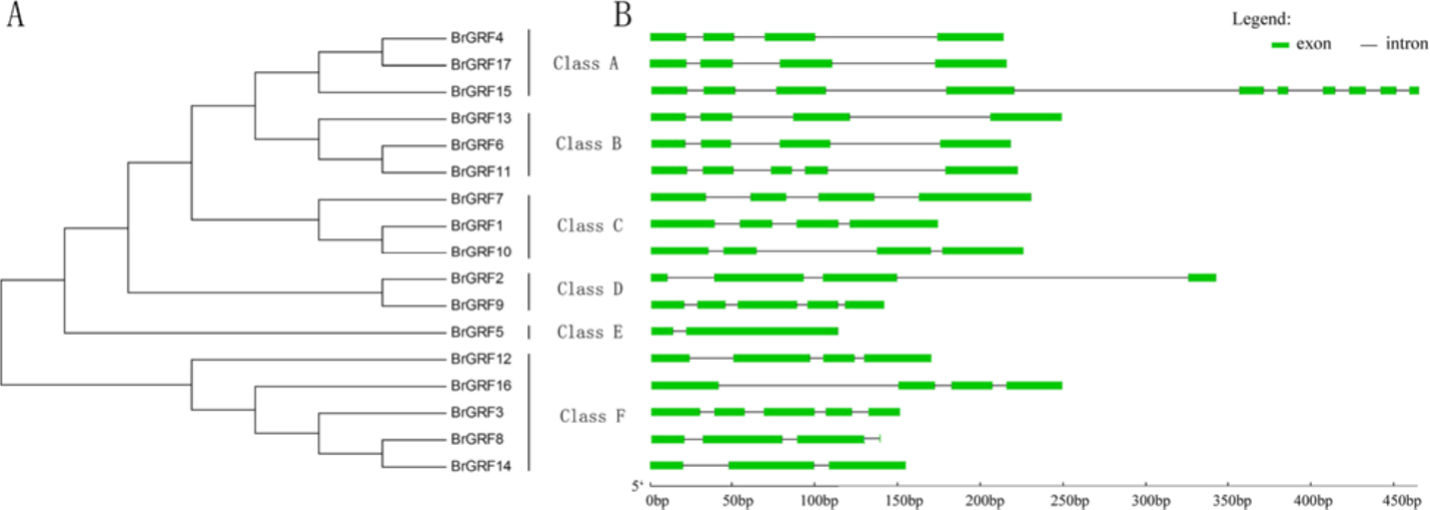
**Chinese cabbage** ***BrGRF*** **gene family. (A)** Phylogenetic relationships among the translated BrGRF proteins. **(B)** Intron/exon structure of the *BrGRF* genes.

Based on the recently sequenced *B. rapa* line Chiifu genome and annotated genes [20], 17 *BrGRF*s were identified from the Brassica database (http://brassicadb.org/brad/) and designated *BrGRF1*–*BrGRF17* according to their distribution in the genome (Table 1). The coding sequence (CDS) lengths of the *BrGRF*s varied widely; *BrGRF15* was the longest (1617 bp) and *BrGRF11* was the shortest (1089 bp). The intron/exon structures of the *BrGRF*s were determined by aligning the CDSs to the genomic sequence. The results indicated that all the *BrGRF* gene sequences contained introns in their CDSs. The number of introns varied from one to nine (Figure 1B); however, most of the genes (10 out of 17) had three introns, followed by four introns (3 out of 17), two introns (2 out of 17), and one and nine introns (1 each out of 17). This result was similar to the findings from previous studies on Arabidopsis (a dicot) where most *AtGRF*s contained three introns (7 out of 9) [4], and on rice (a monocot) where most *OsGRF*s had two introns (6 out of 12), followed by three introns (4 out of 12), and four introns (2 out of 12). Additionally, the number of introns in the CDSs of GRF genes in the same subfamily was different. For example, *BrGRF4*, *BrGRF15*, and *BrGRF17*, which belong to subfamily A (Figure 1A), had three, nine, and three introns respectively.

**Table 1:** **Chinese cabbage** ***BrGRF*** **gene family**

Gene	Accession no.	Chr. (strand)	Start/stop codon	CDS (bp)	GC content (%)	Length ^a^ (aa)	MW ^a^ (kDa)	pI ^a^
*BrGRF1*	Bra011781	A01 (+)	585376/587110	1356	47.49	451	49.44	9.29
*BrGRF2*	Bra013767	A01 (+)	7684376/7685880	1116	47.13	371	40.90	6.44
*BrGRF3*	Bra021521	A01 (-)	24798925/24802342	1236	42.23	411	46.77	7.38
*BrGRF4*	Bra028541	A02 (-)	969253/971390	1101	46.50	366	40.79	8.86
*BrGRF5*	Bra022667	A02 (-)	8154404/8155533	1044	47.51	347	37.98	8.67
*BrGRF6*	Bra023066	A03 (-)	8479530/8481703	1107	50.77	368	40.35	8.70
*BrGRF7*	Bra000575	A03 (-)	11850224/11852523	1557	48.43	518	55.45	9.03
*BrGRF8*	Bra001532	A03 (+)	16888039/16889424	1092	43.59	363	41.14	8.69
*BrGRF9*	Bra019244	A03 (+)	25506702/25508111	1128	48.23	375	40.72	6.62
*BrGRF10*	Bra017851	A03 (-)	30925406/30927659	1344	47.99	447	48.57	9.29
*BrGRF11*	Bra017240	A04 (-)	15818605/15820821	1089	50.23	362	40.01	6.76
*BrGRF12*	Bra039334	A04 (+)	18570071/18571766	1281	45.43	426	47.79	9.32
*BrGRF13*	Bra005268	A05 (+)	4422082/4424566	1167	50.99	388	42.73	8.25
*BrGRF14*	Bra027384	A05 (-)	20638410/20639958	1176	43.11	391	44.08	8.23
*BrGRF15*	Bra019640	A06 (-)	5345436/5350074	1617	43.66	538	60.82	8.25
*BrGRF16*	Bra015184	A07 (-)	2559826/2562310	1203	43.72	400	45.76	9.01
*BrGRF17*	Bra006956	A09 (-)	26481602/26483701	1149	46.56	382	42.86	6.81

^a^Length, MW, and pI refer to the translated BrGRF proteins.

Simple sequence repeat (SSR) markers are useful for a variety of applications in plant genetic mapping and molecular breeding because of their genetic codominance, abundance, dispersal throughout the genome, multi-allelic variation, high reproducibility, and high level of polymorphisms [21]. In this study, 16 SSR markers, including nine di-, six tri-, and one hexa-nucleotide motifs were detected in the 17 identified *BrGRF*s using the online SSR identification tool SSRIT (Table 2). Further, *BrGRF10*, *BrGRF13*, *BrGRF15*, and *BrGRF16* had two SSR markers each, *BrGRF2*–*BrGRF6*, *BrGRF11*, *BrGRF12*, and *BrGRF17* genes had only one SSR marker each, and *BrGRF1*, *BrGRF7*–*BrGRF9*, and *BrGRF14* genes had no SSR markers. Of the detected SSRs in these genes, seven were detected in exons and nine were detected in introns.

**Table 2:** **Simple sequence repeats (SSRs) predicted in the** ***BrGRF*** **s**

Gene	Motif	No. of repeats	SSR start	SSR end	Length ^a^	Intron/exon
*BrGRF2*	cta	10	641	670	3418	exon
*BrGRF3*	cat	5	103	117	1505	exon
*BrGRF4*	ag	13	1383	1408	2138	intron
*BrGRF5*	tcatcc	4	87	110	1130	exon
*BrGRF6*	tc	6	487	498	2174	intron
*BrGRF10*	gct	5	1572	1586	2254	exon
*BrGRF10*	cat	6	1959	1976	2254	exon
*BrGRF11*	caa	5	1806	1820	2217	exon
*BrGRF12*	ca	6	395	406	1696	intron
*BrGRF13*	tc	6	504	515	2485	intron
*BrGRF13*	ca	6	1800	1811	2485	intron
*BrGRF15*	tc	6	517	528	4639	intron
*BrGRF15*	at	7	3723	3736	4639	intron
*BrGRF16*	ta	11	1354	1375	2485	intron
*BrGRF16*	tc	13	1462	1487	2485	intron
*BrGRF17*	gaa	5	1969	1983	2160	exon

^a^Length of the gene from the start codon to the stop codon in the genomic sequence. SSRs were identified using SSRIT (http://archive.gramene.org/db/markers/ssrtool).

**Figure 2:**
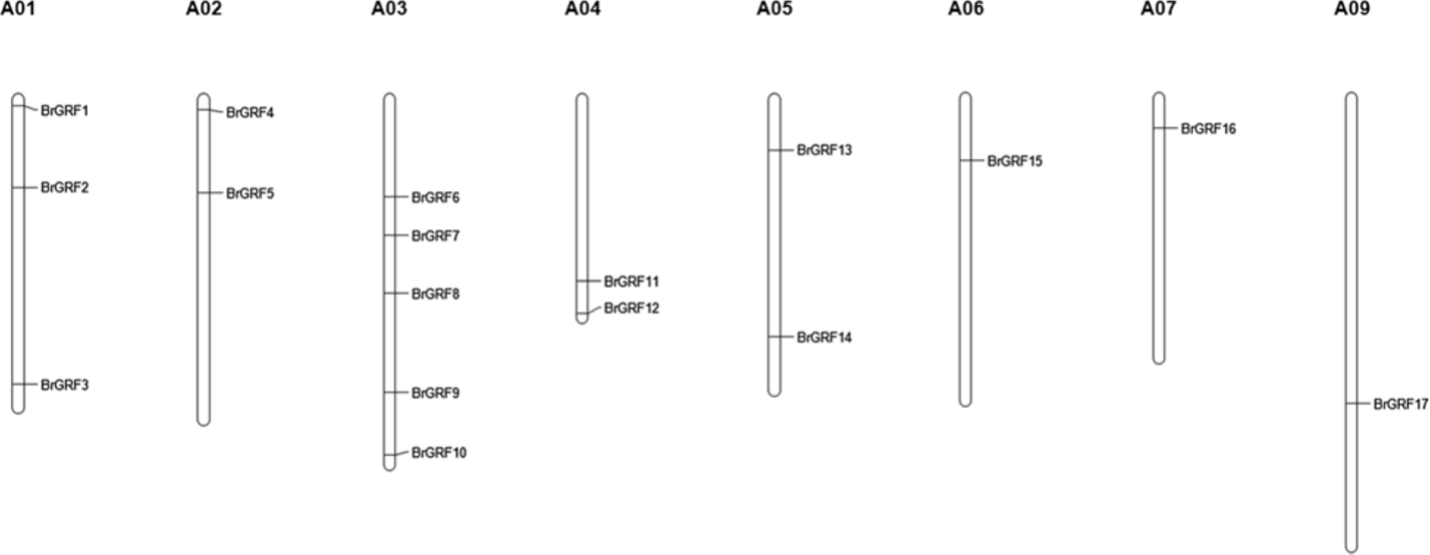
**Locations of the** ***BrGRF*** **genes on the Chinese cabbage chromosomes.** The chromosome number is indicated at the top of each chromosome representation.

The *BrGRF*s were unevenly distributed across the chromosomes (Figure 2), which is similar to previous results in Arabidopsis and rice [4]. The chromosome 03, 01, 02 and 04 has five, three, two and two genes, respectively, whereas the chromosome 06, 07 and 09 each includes only one.

### Conserved domains and motifs in the predicted BrGRF proteins

**Figure 3:**
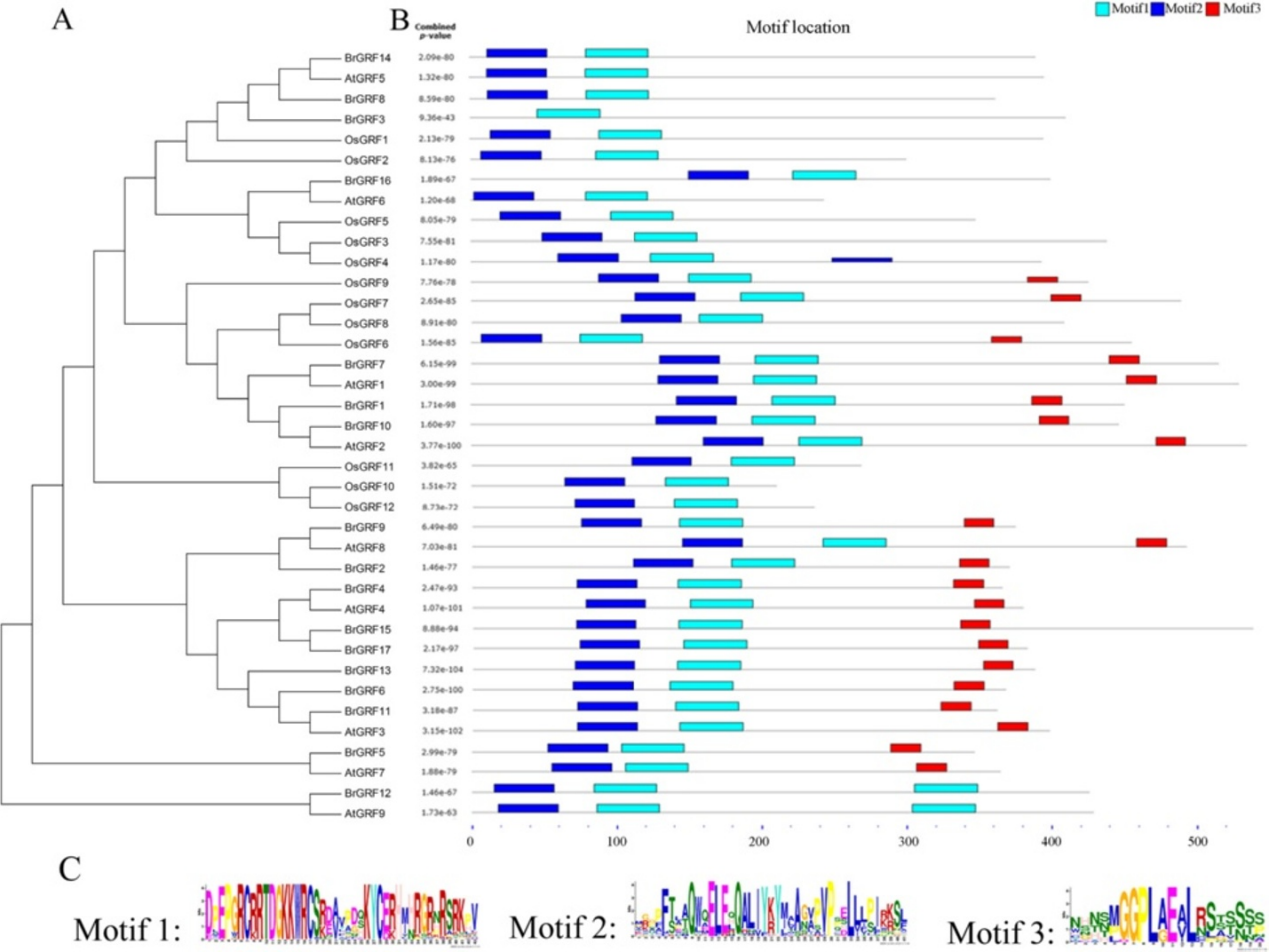
**Conserved domains and motifs in BrGRF proteins. (A)** Phylogenetic tree of Chinese cabbage, rice, and Arabidopsis GRF proteins. **(B)** Distribution of conserved motifs in Chinese cabbage, rice, and Arabidopsis GRF proteins. The GRF protein sequences of Chinese cabbage, Arabidopsis, and rice were obtained from the BRAD (http://brassicadb.org/brad/), TAIR (http://www.arabidopsis.org/) and TIGR (http://www.tigr.org/) databases, respectively. **(C)** Sequence logos of the predicted domains in the BrGRF protein sequences.

**Figure 4:**
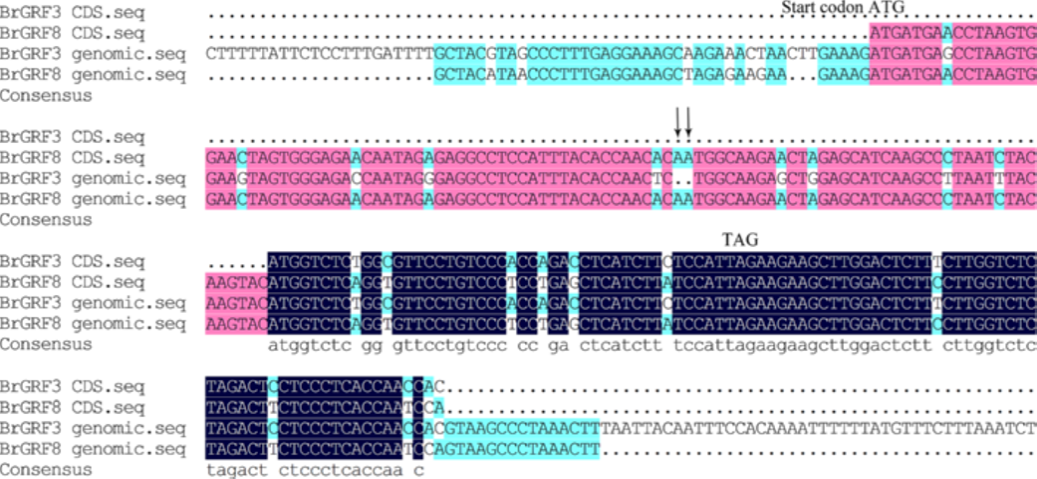
**Multiple sequence alignment of the CDS and genomic sequences of** ***BrGRF3*** **and** ***BrGRF8*** **.**

The modular structures of GRF proteins have been studied thoroughly in Arabidopsis [3], rice [4], maize [5], and *Brachypodium distachyon* [6]. Based on this information, we used the MEME web server (http://meme.nbcr.net/meme/cgi-bin/meme.cgi) to analyze the domain distribution in BrGRFs. Motif 2, specified as the QLQ domain, was predicted to be present in 16 of the 17 BrGRFs, BrGRF3 was the exception (Figure 3). We found that although *BrGRF3* could encode the QLQ domain, the deletion of two adenine residues at positions 62 and 63 bp in the *BrGRF3* sequence compared with the *BrGRF8* sequence caused a frameshift that introduced a TAG stop codon that truncated the BrGRF3 protein sequence (Figure 4). Motif 1, specified as the WRC domain, was predicted in all 17 BrGRF proteins. The results also indicated that AtGRF9 and BrGRF12 contained a second motif 1 downstream of the first. Additionally, motif 3, specified as the GGPL domain, was observed in 12 of the 17 BrGRF proteins, including BrGRF1, BrGRF2, BrGRF4–BrGRF7, BrGRF9–BrGRF11, BrGRF13, BrGRF15, and BrGRF17 (Figure 3).

### Phylogenetic relationships and duplication events of the *BrGRFs*

Classifying genes and constructing a phylogeny are important for the functional analysis of a gene family. A phylogenetic tree of the 17 predicted BrGRFs, 12 rice GRFs (OsGRF) and nine Arabidopsis GRFs (AtGRF) was constructed using MEGA4.1 with the bootstrap–neighbor-joining method. The 17 BrGRF proteins were classified into six subgroups: A, BrGRF1, 7, and 10; B, BrGRF2 and 9; C, BrGRF4, 6, 11, 13, 15, and 17; D, BrGRF5; E, BrGRF12; F, BrGRF3, 8, 14 and 16 (Figure 1A). The phylogenetic tree suggested that the BrGRF proteins were closest to the AtGRF proteins and more distant from the OsGRF proteins (Figure 3A). The phylogenetic tree also indicated a number of putative homologous pairs: BrGRF14/AtGRF5 (identity 82.84%), BrGRF16/AtGRF6 (identity 46.42%), BrGRF7/AtGRF1 (identity 85.05%), BrGRF10/AtGRF2 (identity 66.48%), BrGRF9/AtGRF8 (identity 56.54%), BrGRF4/AtGRF4 (identity 79.79%), BrGRF11/AtGRF3 (identity 77.04%), BrGRF5/AtGRF7 (identity 70.70%), and BrGRF12/AtGRF9 (identity 70.09%) (Figure 3A). Additionally, by comparing the MEME and phylogenetic tree analyses, we found that these homologous pairs usually had similar motif structures; for example, the BrGRF12/AtGRF9 pair had two WRC domains in their N-terminus (Figure 3A and 3B).

Analyzing the duplication events that may have occurred in the Chinese cabbage genome during evolution will help in understanding the evolutionary mechanisms of the *BrGRF*s. Paralogous relationships for these *BrGRF*s have been displayed in http://brassicadb.org/brad/. The results indicated that there were three set of triplicated genes in the Chinese cabbage genome: *BrGRF3*, *8*, and *14* in the F block; *BrGRF6*, *11*, and *13* in the J block; and *BrGRF15*, *4*, and *17* in the N block (Table 3). The other *BrGRF* genes were either duplicated or singletons. No tandem duplication event was detected in the *BrGRF* family.

**Table 3:** **Syntenic** ***GRF*** **genes between Arabidopsis and Chinese cabbage**

tPCK Chr ^a^	Block	Arabidopsis gene	Chinese cabbage gene		
			LF ^b^	MF1 ^c^	MF2 ^c^
tPCK2	F	*AtGRF5* (AT3G13960)	*BrGRF14*	*BrGRF3*	*BrGRF8*
tPCK2	G	*AtGRF6* (AT2G06200)	*BrGRF16*	-	-
tPCK3	J	*AtGRF3* (AT2G36400)	*BrGRF13*	*BrGRF11*	*BrGRF6*
tPCK3	J	*AtGRF9* (AT2G45480)	-	*BrGRF12*	-
tPCK3	I	-	-	-	*BrGRF7*
tPCK4	U	*AtGRF2* (AT4G37740)	*BrGRF1*	*BrGRF10*	-
tPCK4	U	*AtGRF8* (AT4G24150)	*BrGRF2*	*BrGRF9*	-
tPCK5	Wb	*AtGRF7* (AT5G53660)	-	-	*BrGRF5*
tPCK6	N	*AtGRF4* (AT3G52910)	*BrGRF17*	*BrGRF15*	*BrGRF4*

The data were downloaded from the Brassica Database (http://brassicadb.org/brad/).

^a^ *tPCK Chr*, Chromosome of translocation the Proto-Calepineae Karyotype, the ancestral karyotype of the Brassicaceae family. ^b^ *LF*, Less fractioned subgenome. ^c^ *MF1 and MF2*, More fractioned subgenomes.

### Putative functional analysis of the BrGRF proteins

The Gene Ontology (GO) database (http://www.geneontology.org/) is an international standardized gene functional classification system that offers a dynamically updated controlled vocabulary that comprehensively describes the properties of genes and their products in any organism under three main categories: biological process, molecular function, and cellular component. In this study, the 17 predicted BrGRFs were assigned GO terms. The results indicated that most of BrGRFs (except BrGRF3, which had no GO hits) were annotated with terms in the same GO groups, including ATP binding; hydrolase activity, acting on acid anhydrides, in phosphorus-containing anhydrides under the molecular function category, regulation of transcription under the biological process category, and nucleus under the cellular component category (Additional file 1). These data implied that these BrGRF proteins could have similar biological functions. BrGRF15 was also annotated with terms related to hydrolase activity, acting on ester bonds; nuclease activity; DNA binding; recombinase activity under the molecular function category, and response to DNA damage stimulus; DNA recombination; DNA repair; nucleobase, nucleoside, nucleotide and nucleic acid metabolic process under the biological process category. This result suggested that BrGRF15 may be involved in nucleic acid metabolic processes.

### Expression patterns of the *BrGRF*s and *BrGIF*s (GRF interacting factor genes)

**Figure 5:**
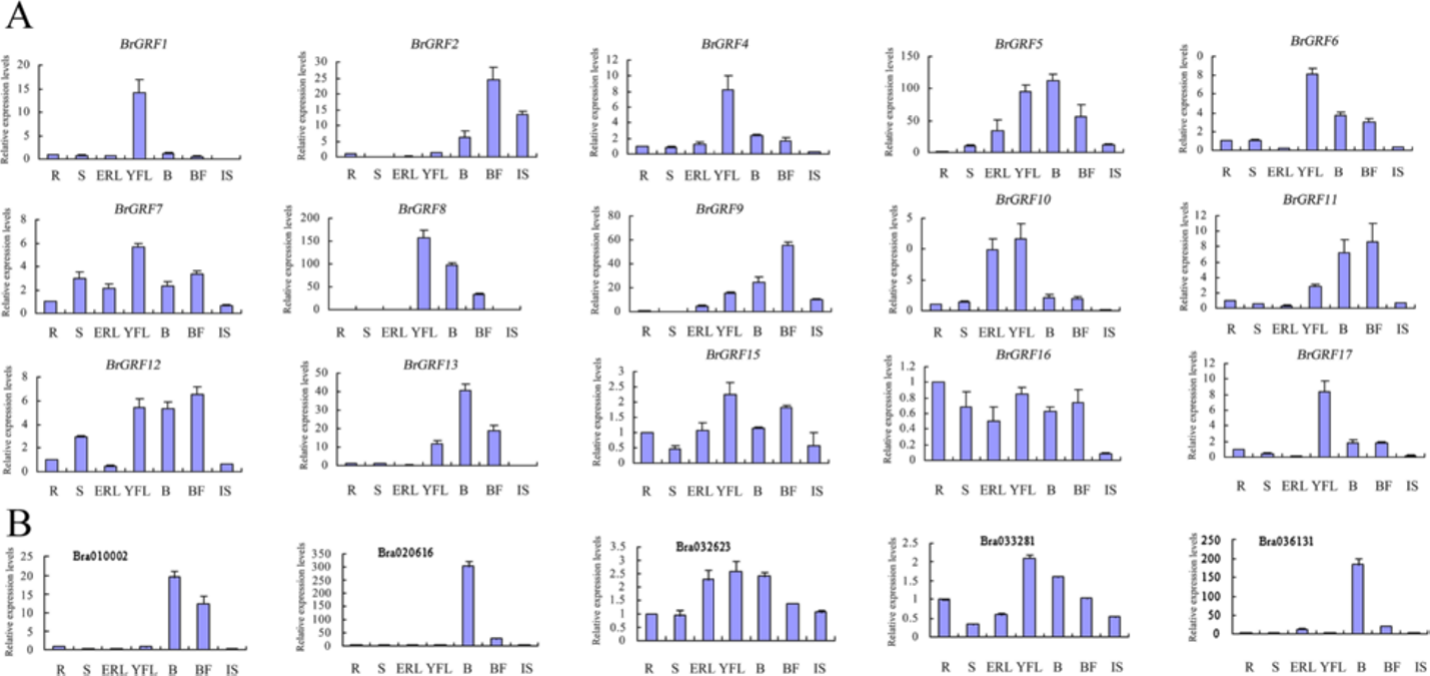
**Expression profiles of the** ***BrGRF*** **(A) and** ***BrGIF*** **(B) genes in organs/tissues of Chinese cabbage.** Samples were collected from roots (R), stems (S), expanded rossete leaves (ERL), young folding leaves (YFL) beginning to fold at the early folding stage (about 24–25 leaves), buds (B), blooming flowers (BF), and immature siliques (IS) 15 d after fertilization. The analysis was carried out by RT-qPCR.

GRF proteins play important roles in the growth and development in plants [2, 3, 7–9, 17–19]. To understand which GRF genes may be involved in regulating specific tissue or organ growth in Chinese cabbage, the expression patterns of the *BrGRF*s in various tissues were investigated by real-time quantitative PCR (RT-qPCR). The results indicated that all 17 *BrGRF*s had higher expression levels in young leaves compared with in old leaves, except for the *BrGRF3* and *BrGRF14*, which were undetectable in all the tissues examined (Figure 5A). In addition, *BrGRF1*, *4*, *5*, *6*, *8*, and *13* had higher expression levels in buds than in blooming flowers, whereas *BrGRF2*, *7*, *9*, *11*, *12*, *15*, and *16* had higher expression levels in blooming flowers than in buds. The expression levels of *BrGRF16* were highest in root tissues. The *BrGRFs* that were expressed mainly in certain organs or tissues might play important roles in the growth and development of these organs or tissues.

**Figure 6:**
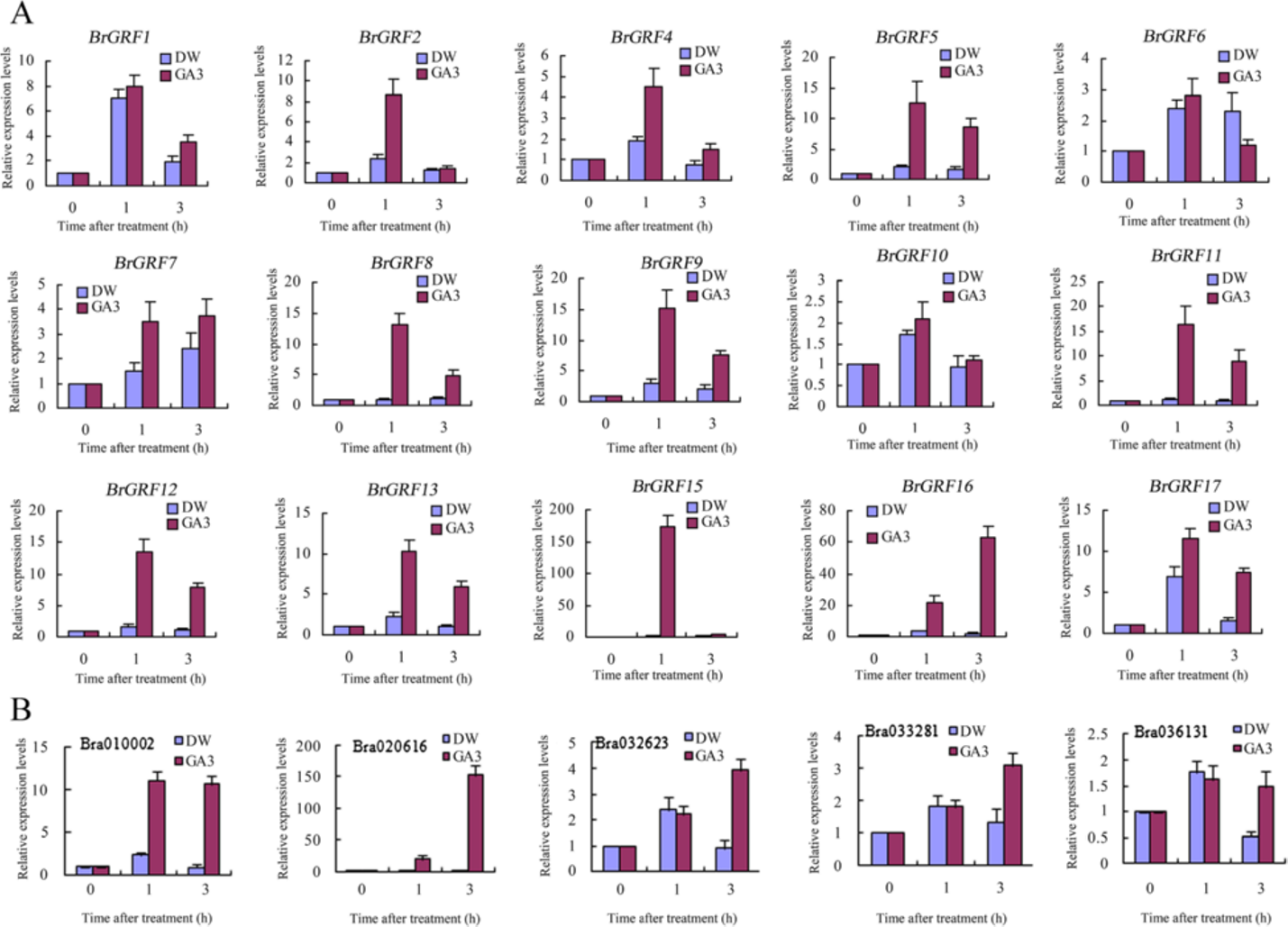
**Gene expression of the** ***BrGRF*** **s (A) and** ***BrGIFs*** **(B) in response to GA3 treatment.** GA3, 100 μM gibberellic acid treatment; DW, distilled water treatment as the control. The analysis was carried out by RT-qPCR.

The response to GA3 treatment was also investigated. Compared with distilled water (DW) treatment, the transcript levels of *BrGRF5*, *8*, *9*, *11*, *12*, *13*, *15*, *16*, and *17* were increased more than 5-fold and *BrGRF2*, *4*, and *7* were increased 2- to 5-fold in response to 100 μM GA3 application (Figure 6A). The expression levels of the other *BrGRF*s were only slightly affected (less than 2-fold) or not affected by the GA3 treatment.

GRF and GIF proteins may act as transcription activators or coactivators, respectively, as part of a complex involved in the growth and development in plants [4, 22]. Therefore, the expression patterns of *BrGIF* genes, including *Bra010002*, *Bra020616*, *Bra036131*, *Bra032623* and *Bra033281* were also investigated by RT-qPCR.

The results indicated that the *Bra010002*, *Bra020616*, and *Bra036131* genes had the highest expression levels in buds, while *Bra032623* and *Bra033281* had the highest expression levels in young leaves (Figure 5B). Additionally, the transcript expression levels of *BrGIFs* could be increased by GA3 treatment. At 3 h after GA3 treatment, the transcript levels were 11.9-, 199.6-, 4.3-, 2.3-, and 2.8-fold higher for *Bra010002*, *Bra020616*, *Bra032623*, *Bra033281* and *Bra036131*, respectively, compared with their levels after DW treatment (Figure 6B).

### Ectopic expression of *BrGRF8*in Arabidopsis increases organ size

**Figure 7:**
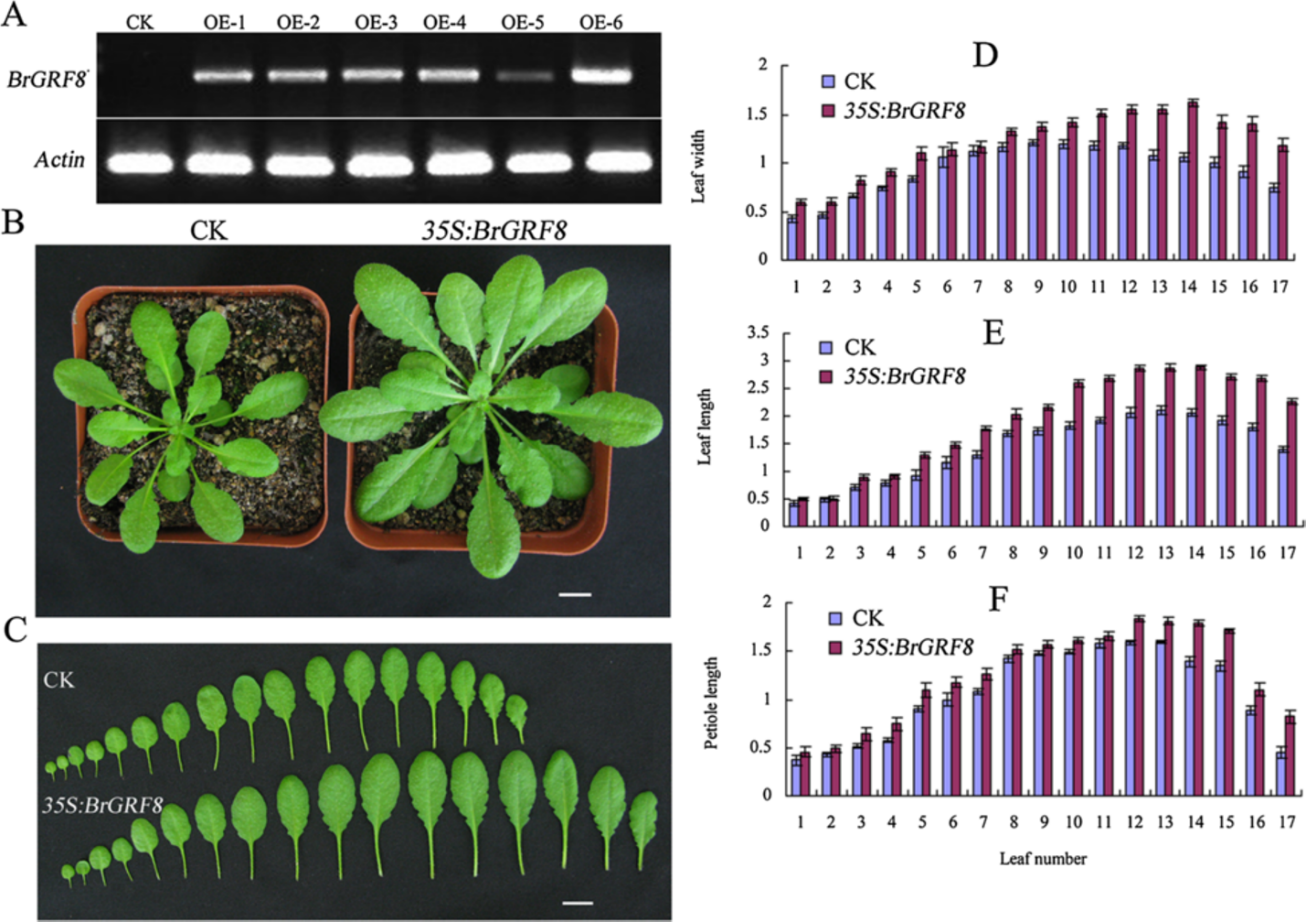
**Ectopic expression of** ***BrGRF8*** **in Arabidopsis. (A)** RT-PCR analysis of *BrGRF8* expression in different transgenic Arabidopsis lines. **(B)** 40-day-old vector control (CK) and *35S:BrGRF8* transgenic plants grown under the same conditions. Scale bar: 1 cm. **(C)** The first 17 leaves of 40-day-old vector control (CK) and *35S:BrGRF8* transgenic plants. Scale bar: 1 cm. Leaf width **(D)**, leaf length **(E)**, and petiole length **(F)** of 40-day-old vector control (CK) and *35S:BrGRF8* transgenic plants.

**Figure 8:**
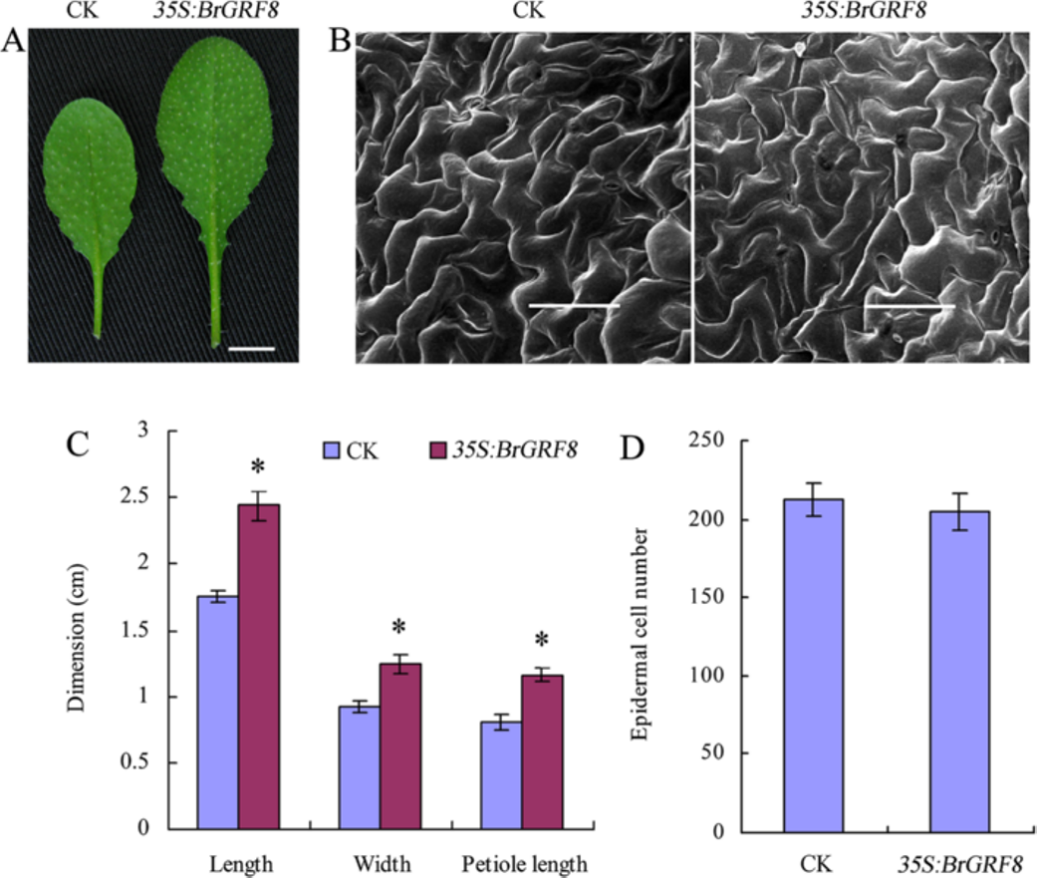
**Morphology and histological analysis of fifth rosette leaves of 35-day-old plants. (A)** Morphology of the fifth rosette leaves of vector control (CK) and *35S:BrGRF8* transgenic plants. Scale bar: 1 cm. **(B)** Scanning electron micrographs of the fifth rosette leaves of vector control (CK) and *35S:BrGRF8* transgenic plants. Scale bars: 100 μm. **(C)** Dimensions of the fifth rosette leaves. Error bars represent ± SD (*n* = 10). The star (*) indicate values that were significantly different (T-test; *p* <0.05). **(D)** Number of epidermal cells per unit area (mm^2^) in the adaxial surface of fully expanded fifth rosette leaves of vector control (CK) and *35S:BrGRF8* transgenic plants. The middle areas of a half leaf beside the midvein were examined. Error bars represent ± SD (*n* = 5).

Compared with the other *BrGRF*s, *BrGRF8* had the highest transcription level ratio between immature and mature leaves, implying that it might play an important role in the growth and development of Chinese cabbage leaves. To explore the functions of *BrGRF8*, we examined the Arabidopsis phenotypes caused by the ectopic expression of this gene. The transgenic line developed larger leaves and other organs compared with the vector control lines. Quantitatively, the dimensions of each of the first 17 leaves (in each plant), including leaf width, leaf length, and petiole length, increased dramatically in the *35S:BrGRF8* transgenic plants (Figure 7) compared with their dimensions in the vector control plants. However, there was little difference between the *35S:BrGRF8* and vector control plants in terms of silique size and seed number per silique (Table 4). The increase in leaf area in the *BrGRF8* over-expressers was mediated directly by an increase in cell number because the adaxial epidermal surface cell sizes were normal (Figure 8).

**Table 4:** **Morphology of the** ***35S:BrGRF8*** **transgenic Arabidopsis plants**

Variable	Vector control (n)	*35S:BrGRF5*(ncpa)
Hypocotyl length (mm)^a^	11.47 ± 1.06 (15)	15.40 ± 1.30^*^ (15)
Seedling length (mm)^b^	21.55 ± 3.50 (10)	29.41 ± 2.69^*^ (10)
Silique length (mm)	12.43 ± 1.00 (20)	12.73 ± 1.13 (20)
Seeds/silique	47.45 ± 2.06 (20)	48.15 ± 1.79 (20)

The measured values are given as mean ± standard error and ‘n’ indicates the number of plants per genotype. The star (*) indicate values that were significantly different (T- test; *p* <0.05). ^a^Seedlings grown in the dark for 6 days. ^b^Root length plus hypocotyl length of 20-day-old seedlings grown at 22°C under a 16/8-h (light/dark) photoperiod.

## Discussion

Previous studies have shown that GRF genes have positive functions in regulating organ size by promotion and/or maintenance of cell proliferation activity [2, 7, 8]. However, it is not known whether or how GRF proteins in Chinese cabbage regulate organ size. Because GRF proteins in different species may have different regulatory roles during the process of plant development, it is necessary to study the GRF genes in each species to understand the mechanisms of plant organ size control. In this study, a comprehensive set of 17 non-redundant GRF proteins were identified and characterized. Although the *B. rapa* genome is approximately 4-fold larger than the Arabidopsis genome (485 Mb and125 Mb, respectively), the gene number in *B. rapa* is only twice that of Arabidopsis (17:9), suggesting that there was extensive gene loss during genome duplication [20, 23].

Pseudogenes are non-functional copies of gene fragments incorporated into the genome either by retrotransposition of mRNA or by duplication of genomic DNA. Pseudogenes are widely distributed in eukaryotic genomes. Sequence alignment showed that the *BrGRF3* and *BrGRF8* gene sequences were highly similar, and that there was a deletion of two A residues in the *BrGRF3* CDS compared with the *BrGRF8* CDS that lead to the introduction of a premature stop codon. Because, *BrGRF3* gene expression was not detected in the various tissues, even after GA3 treatment, we suggest that *BrGRF3* may be a pseudogene in the Chinese cabbage genome.

Phylogenetic analysis of the predicted GRF protein sequences from Chinese cabbage, Arabidopsis, and rice indicated that the BrGRFs shared higher similarity with AtGRFs than with OsGRFs, which is consistent with their evolutionary relationships: Chinese cabbage and Arabidopsis are both dicots of the *Brassicaceae* family, while rice is a monocot of the *Poaceae* family. Additionally, the phylogenetic and gene duplication analyses revealed that the *BrGRF*s contained three triplets, two duplicates, and four singletons. However, none of the *BrGRF*s exhibited tandem duplication. These results suggest that the expansion of the *BrGRF*s could be explained by the ancestor genome of *B. rapa* experiencing a whole-genome triplication and evolving finally into Chinese cabbage [20, 23].

The *BrGRF*s were expressed mainly in specific organs and tissues, suggesting that they may play important roles in the growth and development of these organs or tissues. We found that the transcript levels of the *BrGRF1*, *4*, *6*, *8*, *10*, *15*, and *17* genes were higher in young leaves than in the other tested tissues, suggesting that these genes are involved mainly in the growth and development of young leaves in Chinese cabbage. Thus, improving the expression levels of these genes might help improve Chinese cabbage leaf head yield.

GA3 is involved in various physiological processes in plants and KNOX proteins contribute to the regulation of meristem maintenance by negatively regulating the production of gibberellins [11–16]. Therefore, downregulation of *KNOX* gene expression at the flanks of the SAM cycle was reported to increase the level of GA, resulting in organized cell proliferation and determination of cell fate [13]. A previous study showed that GRF proteins act as repressors and down-regulators of *KNOX* gene expression [10]. As reported previously in rice [4], the transcription of most *BrGRF*s was induced by GA3 treatment. These results suggested that the GRF genes may function in maintaining or promoting cell proliferation in plants by a feedback regulation mechanism, in which the GRF genes positively regulate the production of gibberellins, and GAs in turn upregulate GRF gene expression.

In previous studies, the over-expression of GRF genes in Arabidopsis was found to result in bigger organ size than the vector control [3, 7], while reduction of *AtGRF* gene expression by the overexpression of the microRNA miR396 in transgenic Arabidopsis caused narrow-leaf phenotypes due to a reduction in cell number [24–26]. Here, we found that the ectopic expression of *BrGRF8* also increased leaf size in Arabidopsis. In addition, our histological results revealed a significant increase in cell number but not in cell size in the *35S:BrGRF8* transgenic Arabidopsis plants compared with the vector control plants, suggesting that *BrGRF8* may control the growth of plant organs by regulating cell proliferation rather than by enlarging cell volume.

## Conclusions

We identified 17 members of the Chinese cabbage GRF gene family that encoded putative GRF proteins that fell into six subfamilies. The phylogenetic relationships among Chinese cabbage, rice, and Arabidopsis GRF genes, suggested that the *BrGRF*s were more closely allied with *AtGRF*s than with *OsGRF*s. Further, phylogenetic and duplication event analysis suggested that whole genome duplication may have been the main contributor to the expansion of the *BrGRF*s. Additionally, the ectopic expression of *BrGRF8* in Arabidopsis positively controlled organ size by regulating cell proliferation. The expression profiles obtained by RT-qPCR showed that the *BrGRF*s may be involved in immature organ or tissue growth and development via the GA pathway. Together, these data will not only contribute to a further understanding of the characteristics and functions of the GRF family in different species, but will also provide a promising strategy for Chinese cabbage breeding programs to improve yield/head size.

## Methods

### Identification and analysis of GRF genes in Chinese cabbage

The nucleotide and protein sequences of BrGRFs were identified based on the *B. rapa* line Chiifu genome sequence (http://brassicadb.org) [20]. The GRF nucleotide sequences were aligned using DNAMAN 6.0.40 (Lynnon Biosoft, USA). Intron/exon structure analysis was performed using the Gene Structure display Server (GSDS) (http://gsds.cbi.pku.edu.cn/). Phylogenetic trees were constructed with the MEGA 4.0 software using the neighbor-joining method and a bootstrap test that was replicated 1000 times [27]. The GC content was calculated by DNASTAR (Madison, WI, USA). The number of amino acids, molecular weight (MW), and theoretical isoelectric point (pI) were computed using the ProtParam tool (http://web.expasy.org/protparam/). The SSR markers were detected using the SSRIT software (http://archive.gramene.org/db/markers/ssrtool) with the parameters adjusted for identification of perfect di-, tri-, tetra-, penta-, and hexa-nucleotide motifs with a minimum of 6, 5, 5, 4 and 4 repeats, respectively. The GO analysis of BrGRF proteins was carried out using the GO term analysis tool in Gramene (http://www.geneontology.org/) developed by the GO consortium Conserved motifs in the full-length amino acid sequences of GRF proteins from Chinese cabbage, Arabidopsis, and rice were identified using MEME [28]. The Arabidopsis and rice GRF protein sequences were downloaded from the Arabidopsis Information Resource (TAIR: http://www.arabidopsis.org/) and the Institute for Genomic Research Rice Genome Annotation project (TIGR: http://www.tigr.org/), respectively.

### Plant materials and GA3 treatment

Chinese cabbage (*Brassica rapa* L. ssp. *pekinensis* inbred line Fushanbaotou) plants were grown in a greenhouse at 20 ± 2°C with a photoperiod of 16 h light and 8 h dark. For the GA3 treatment, uniformly sized seedlings were selected when they had developed four fully opened leaves, and then treated with 100 μM GA3 or distilled water (DW). The leaves of the seedlings were harvested after 0, 1, and 3 h of GA3 treatment. For analysis of GRF genes expression in different tissues, roots (R), stems (S), expanded rossete leaves (ERL), young folding leaves (YFL) beginning to fold at the early folding stage (about 24–25 leaves), buds (B), blooming flowers (BF), and immature siliques (IS) 15 d after fertilization were collected. All materials were frozen immediately in liquid nitrogen, and stored at -80°C until RNA isolation.

### Real-time quantitative PCR

Total RNA was extracted from each sample using Trizol reagent (Invitrogen, Carlsbad, CA, USA) and treated with RNase-free DNase I (Takara, Dalian, China) for 45 min according to the manufacturer’s protocol. First-strand cDNA was synthesized from 1 μg of total RNA using a PrimeScript 1^st^ Strand cDNA Synthesis Kit (Takara). RT-qPCR was carried out using a SYBR Green Master mix (Takara) on an IQ5 Real-Time PCR Detection System (Bio-Rad, Hercules, CA, USA). The gene-specific primers designed for the *BrGRF* and *BrGIF* genes are listed in Additional file 2. The actin gene was used as a constitutive expression control in the RT-qPCR experiments. The PCR cycling conditions comprised an initial polymerase activation step of 95°C for 1 min, followed by 40 cycles of 95°C for 10 s and 60°C for 30 s. After each PCR run, a dissociation curve was designed to confirm the specificity of the product and to avoid the production of primer dimers. The relative amounts of the amplification products were calculated by the comparative 2^-ΔΔCт^ method [29].

### Transformation of Arabidopsis

To create transgenic plants overexpressing *BrGRF8*, the full-length cDNA was amplified by PCR with the primers BrGRF8-forward (5′-CG*GGATCC*ATGATGAACCTAAGTGGAACTAGTG-3′) and BrGRF8-reverse (5′-TA*GTCGAC*TCAGCTACCAGTGTCGAGTCTTGAC-3′). The *underlined sequences* represent restriction sites for *Bam*HI and *Sal*I, respectively. The amplified DNA fragments were cloned into the pGEM-T easy vector (Promega, Madison, WI, USA) and sequenced using an ABI 3730 × l DNA Sequencer (Applied Biosystems, Foster City, CA, USA). Then, the *BrGRF8* cDNA in the pGEM-T easy vector was double-digested with *BamHI* and *SalI* and subcloned downstream of the CaMV 35S promoter into the binary vector pCAMBIA2300-35S-OCS to construct the plasmid CaMV35S*:BrGRF5* (*35S:BrGRF8*). The pCAMBIA2300-35S-OCS vector without the *BrGRF8* cDNA insert was used as a negative vector control. The *35S:BrGRF8* and vector control constructs were transformed separately into *Agrobacterium tumefaciens* LBA4404 and then the *A. tumefaciens*-mediated transformation of Arabidopsis was performed via vacuum infiltration [30]. The transgenic plants were screened on 1/2 MS agar plates containing 50 mg/ml kanamycin sulfate, and further verified by reverse transcription PCR. Homozygous T3 generation transgenic plants were used for subsequent analyses.

### Histological analysis

Fully expanded fifth leaves of the vector control and *35S:BrGRF8* plants were detached and fixed in 100 mM sodium phosphate buffer (pH 7.0) containing 4% glutaraldehyde for 2 h at room temperature. Following a brief rinse in the buffer, the samples were dehydrated in an ethanol series for 1 h at each gradation. The dehydrated samples were dried in a critical-point dryer with liquid CO_2_ as the transitional fluid, and examined using a scanning electron microscope (SEM; JEOL JSM-7600 F, Japan) after coating the samples with gold. Epidermal cell size and number of cells on the adaxial side were determined in the middle region of a half leaf near the midvein. At least five leaves from each of the transgenic plants were selected for counting epidermal cell number in a fixed area on the SEM images.

## Electronic supplementary material

Additional file 1: **Putative functions and cellular localizations of GRF proteins in Chinese cabbage.** (DOC 38 KB)

Additional file 2: **Primers used in real-time quantitative PCR.** (DOCX 15 KB)
