# Supplementary material for: Genome-wide identification and analysis of the growth-regulating factor family in Chinese cabbage (Brassica rapa L. ssp. pekinensis)
Source: BMC Genomics. 2014 Sep 22;15(1):807. doi: 10.1186/1471-2164-15-807 (PMC4180144; doi:10.1186/1471-2164-15-807)
Supplement: Supplementary file 1 — Additional file 1: Putative functions and cellular localizations of GRF proteins in Chinese cabbage. (DOC 38 KB) [file 12864_2014_6487_MOESM1_ESM.doc]

Additional file 1 Putative functions and cellular localizations of GRF proteins in Chinese cabbage.

| Gene name | GO: Molecular function | GO: Biological process | GO: Cellular component | |
| --- | --- | --- | --- | --- |
| BrGRF1  BrGRF2  BrGRF3  BrGRF4  BrGRF5  BrGRF6  BrGRF7  BrGRF8  BrGRF9  BrGRF10  BrGRF11  BrGRF12  BrGRF13  BrGRF14  BrGRF15  BrGRF16  BrGRF17 | ATP binding; Hydrolase activity, acting on acid anhydrides, in phosphorus-containing anhydrides  ATP binding; Hydrolase activity, acting on acid anhydrides, in phosphorus-containing anhydrides  No hit  ATP binding; Hydrolase activity, acting on acid anhydrides, in phosphorus-containing anhydrides  ATP binding; Hydrolase activity, acting on acid anhydrides, in phosphorus-containing anhydrides  ATP binding; Hydrolase activity, acting on acid anhydrides, in phosphorus-containing anhydrides  ATP binding; Hydrolase activity, acting on acid anhydrides, in phosphorus-containing anhydrides  ATP binding; Hydrolase activity, acting on acid anhydrides, in phosphorus-containing anhydrides  ATP binding; Hydrolase activity, acting on acid anhydrides, in phosphorus-containing anhydrides  ATP binding; Hydrolase activity, acting on acid anhydrides, in phosphorus-containing anhydrides  ATP binding; Hydrolase activity, acting on acid anhydrides, in phosphorus-containing anhydrides  ATP binding; Hydrolase activity, acting on acid anhydrides, in phosphorus-containing anhydrides  ATP binding; Hydrolase activity, acting on acid anhydrides, in phosphorus-containing anhydrides  ATP binding; Hydrolase activity, acting on acid anhydrides, in phosphorus-containing anhydrides  ATP binding; Hydrolase activity, acting on acid anhydrides, in phosphorus-containing anhydrides; Hydrolase activity, Acting on ester bonds; Nuclease activity; DNA binding; Recombinase activity  ATP binding; Hydrolase activity, acting on acid anhydrides, in phosphorus-containing anhydrides  ATP binding; Hydrolase activity, acting on acid anhydrides, in phosphorus-containing anhydrides | Regulation of transcription  Regulation of transcription  No hit  Regulation of transcription  Regulation of transcription  Regulation of transcription  Regulation of transcription  Regulation of transcription  Regulation of transcription  Regulation of transcription  Regulation of transcription  Regulation of transcription  Regulation of transcription  Regulation of transcription  Regulation of transcription; Response to DNA damage stimulus; DNA recombination; DNA repair; Nucleobase, nucleoside, nucleotide and nucleic acid metabolic process  Regulation of transcription  Regulation of transcription | Nucleus  Nucleus  No hit  Nucleus  Nucleus  Nucleus  Nucleus  Nucleus  Nucleus  Nucleus  Nucleus  Nucleus  Nucleus  Nucleus  Nucleus  Nucleus  Nucleus |  |
