# Supplementary material for: Genome-wide identification and analysis of the growth-regulating factor family in Chinese cabbage (Brassica rapa L. ssp. pekinensis)
Source: BMC Genomics. 2014 Sep 22;15(1):807. doi: 10.1186/1471-2164-15-807 (PMC4180144; doi:10.1186/1471-2164-15-807)
Supplement: Supplementary file 2 — Additional file 2: Primers used in real-time quantitative PCR. (DOCX 15 KB) [file 12864_2014_6487_MOESM2_ESM.docx]

Additional file 2 Primers used in real-time quantitative PCR

| **Gene** | **Primers（5’-3’）** |
| --- | --- |
| *BrGRF1*  *BrGRF2*  *BrGRF3*  *BrGRF4*  *BrGRF5*  *BrGRF6*  *BrGRF7*  *BrGRF8*  *BrGRF9*  *BrGRF10*  *BrGRF11*  *BrGRF12*  *BrGRF13*  *BrGRF14*  *BrGRF15*  *BrGRF16*  *BrGRF17*  *Bra010002*  *Bra020616*  *Bra032623*  *Bra033281*  *Bra036131*  *Actin* | F: CTTCATTGCTCACATCGTCGTA  R: TGGCTTTGGATCGGTAGGTC  F: CACAATGACACTCGCACCACTAA  R: ATCGTCTTCTTTCATAAACCACC  F: TTCAGATTCTTGGCTTGACCTT  R: TCAGTTTAGCGACTTCTGTAGG  F: CAGATGATGGTAACACCCTGAG  R: TCTTCTACTTTATCGCCTGTGG  F: AGATGGCTTGCTTCCACTTCC  R: TCTTGCTGCTATCATCAGACACT  F: AGGAAGTGGGTCAAGGTGGAT  R: CAAAGAAGTGCCTCAAGATGTG  F: GTCGATTCCTGGACAGAAGAGC  R: GGATAGCAGGGTCAAACTCACG  F: CGAGTTCAACAAGCCCACAAGA  R: TCTGTGCCCAGTCCTCACCAAA  F: AGGAAGGTGAGGGAAGAAGCC  R: GGAGACGCCAAGACAAAGTGC  F: CAAACCGAGAAACAACAACAGC  R: TCACCAGTGGAGGAAGGAGATG  F: TGAAGAGGAAGCCAGGAGCAAT  R: GTTGAAACACCGAGCTGATGGA  F: TGTTAATTGCGGTGGCTTGTTT  R: ACCTTCCTGGTTCGCTGTCTGT  F: CTCTAAAGCTGTCTACAGGCAACG  R: TCTCAGGACTTCGGCTAATGGT  F: TCAAGAAGAGGAGGAGAAAGAT  R: GAAGAAAGGTCAAGCCAAGAAT  F: TTCAGACCCTTCTAATATGATTGC  R: GGATAACCAACTACTAAGCCAGAC  F: TAGAGCCTTCCGGGTCAATCAA  R: GCGTCCCTAACTCAGTGCCAAC  F: CCCTCCCTCAGACGTTTCCTTA  R: CTCCACTGCTCCACCAGTTCAT  F: GGCCGTGGGAAGCCGGAGATGGGTA  R: CTGGACCTTACTTGTGCAACACTCA  F: GGCCGTGGGAAGTTGGAAATGGGA  R: TACTTCATCATACTCTTTTCAGTTC  F: GCCTCCGCAAATGTTTCCGA  R: GGTGTAGCTCCAGCTGTAGA  F: AGCAAATGTTTCCGGCGATG  R: CAGGTGCAGGTGCTGTAGTA  F: CTACCCCAGCAATGTCACCTC  R: CTTCTGCCTGAATAACCCCAC  F: GCTTACGTCGCTCTTGACTACG  R: GATGGTGATGACTTGTCCATCAG |
